# Obesity-Related Genetic Variants and their Associations with Physical Activity

**DOI:** 10.1186/s40798-015-0036-6

**Published:** 2015-10-15

**Authors:** Harold Lee, Garrett I. Ash, Theodore J. Angelopoulos, Paul M. Gordon, Niall M. Moyna, Paul S. Visich, Robert F. Zoeller, Heather Gordish-Dressman, Ved Deshpande, Ming-Hui Chen, Paul D. Thompson, Eric P. Hoffman, Joseph M. Devaney, Linda S. Pescatello

**Affiliations:** 1 Department of Behavioral and Social Sciences, Brown University School of Public Health, Box G-S121-2, Providence, RI 02912 USA; 2 Department of Kinesiology, University of Connecticut, Storrs, CT 06269 USA; 3 Obesity Center & School of Health Sciences, Emory & Henry College, Marion, VA 24354 USA; 4 Department of Health, Human Performance and Recreation, Baylor University, Waco, TX 76798 USA; 5 Department of Sport Science and Health, Dublin City University, Dublin, 7008802 Ireland; 6 Exercise & Sport Performance, University of New England, Biddeford, ME 04005 USA; 7 Department of Exercise Science and Health Promotion, Florida Atlantic University, Boca Raton, FL 33431 USA; 8 Research Center for Genetic Medicine, Children’s National Medical Center, Washington, DC 20010 USA; 9 Department of Statistics, University of Connecticut, Storrs, CT 06269 USA; 10 Division of Cardiology, Henry Low Heart Center, Hartford Hospital, Hartford, CT 06102 USA; 11 Institute for Systems Genomics, University of Connecticut, Storrs, CT 06269 USA

## Abstract

**Background:**

Meta-analysis of genome-wide association studies identified obesity-related genetic variants. Due to the pleiotropic effects of related phenotypes, we tested six of these obesity-related genetic variants for their association with physical activity: fat mass and obesity-associated (*FTO*)(rs9939609)T>A, potassium channel tetramerization domain containing (*KCTD15*) (rs11084753)G>A, melanocortin receptor4 (*MC4R*)(rs17782313)T>C, neuronal growth regulator 1 (*NEGR1*)(rs2815752)A>G, SH2B adapter protein 1 (*SH2B1*)(rs7498665)A>G, and transmembrane protein18 (*TMEM18*)(rs6548238)C>T.

**Method:**

European-American women (*n* = 263) and men (*n* = 229) (23.5 ± 0.3 years, 24.6 ± 0.2 kg/m^2^) were genotyped and completed the Paffenbarger physical activity Questionnaire. Physical activity volume in metabolic energy equivalents [MET]-hour/week was derived from the summed time spent (hour/week) times the given MET value for vigorous, moderate, and light intensity physical activity, and sitting and sleeping, respectively. Multivariable adjusted [(age, sex, and body mass index (BMI)] linear regression tested associations among genotype (dominant/recessive model) and the log of physical activity volume.

**Result:**

*MC4R* (rs17782313)T>C explained 1.1 % (*p* = 0.02), *TMEM18*(rs6548238)C>T 1.2 % (*p* = 0.01), and *SH2B1* (rs7498665)A>G 0.6 % (*p* = 0.08) of the variability in physical activity volume. Subjects with the *MC4R* C allele spent 3.5 % less MET-hour/week than those with the TT genotype (*p* = 0.02). Subjects with the *TMEM18* T allele spent 4.1 % less MET-hour/week than those with the CC genotype (*p* = 0.01). Finally, subjects with the *SH2B1* GG genotype spent 3.6 % less MET-hour/week than A allele carriers (*p* = 0.08).

**Conclusion:**

Our findings suggest a shared genetic influence among some obesity-related gene loci and physical activity phenotypes that should be explored further. Physical activity volume differences by genotype have public health importance equating to 11–13 lb weight difference annually.

## Key Points

- As we hypothesized, the obesity-related SNPs *MC4R* (rs17782313) T>C and *TMEM18* (rs6548238) C>T significantly associated with physical activity volume, while *SH2B1* (rs7498665) A>G trended towards significance.
- *MC4R* (rs17782313) T>C, *TMEM18* (rs6548238) C>T, and *SH2B1* (rs7498665) A>G accounted for ~1 % of the variance in physical activity levels each.
- Our findings have public health significance as the genotype differences in physical activity volume we found ranged from 10.1 to 11.8 MET-hour/week equating to a potential weight differential of 10.8–12.7 lb annually.

## Background

Overweight and obesity are an epidemic affecting more than 68.5 % of U.S. adults [1]. To curb this alarming statistic, a plethora of weight loss strategies have been proposed ranging from counseling [2] and text messaging [3] to incentivizing [4] and pharmaceutical interventions [5]. Many of these strategies have proven efficacious in the short term, but less successful in long-term weight loss and maintenance of that weight loss. Physical activity is not only effective in achieving weight loss, but also essential in predicting successful weight loss maintenance [6, 7]. Therefore, the American College of Sports Medicine (ACSM) recommends 150–250 min/week of moderate intensity physical activity for weight loss, and even greater amounts for weight loss maintenance [8, 9]. However, less than half (49.1 %) of U.S. adults meet the ACSM physical activity recommendations, and 23.7 % of U.S. adults do not participate in any leisure time physical activity [10]. Despite the important role of physical activity in obesity treatment and its promise for long-term weight loss maintenance, it is not clear why some individuals are more likely to participate in habitual physical activity than others to maintain a healthy body weight.

Twin and family studies have shown that genetic predispositions contribute to overweight and obesity [11–15] with heritability accounting for 37–78 % of the variance in obesity-related phenotypes. In 2009, the Genetic Investigation of Anthropometric Traits (GIANT) consortium conducted a meta-analysis of genome-wide association studies (GWAS) involving 32,387 individuals of European ancestry and identified eight genetic variants that associated with body mass index (BMI) [16]. These single nucleotide polymorphisms (SNPs) were fat mass and obesity-associated (*FTO*) (rs9939609) T>A, glucosamine-6 phosphate deaminase 2 (*GNPDA*) (rs10938397) A>G, potassium channel tetramerization domain containing (*KCTD15*) (rs11084753) G>A, melanocortin receptor 4 (*MC4R*) (rs17782313) T>C, mitochondrial carrier homolog 2 (*MTCH2*) (rs10838738) A>G, neuronal growth regulator 1 (*NEGR1*) (rs2815752) A>G, SH2B adapter protein 1 (*SH2B1*) (rs7498665) A>G, and transmembrane protein 18 (*TMEM18*) (rs6548238) C>T.

There is a growing literature showing the important effect-mediation that physical activity has on the genetic predispositions to be obese [15, 17–19]. The genetic variants examined in these studies were mainly those identified in the GWAS by the GIANT consortium to associate with BMI. We are part of an interdisciplinary research team that has completed a large exercise genomics study, Functional Single Nucleotide Polymorphisms Associated with Human Muscle Size and Strength (FAMuSS, NIH R01 NS40606-02). Our colleague, Orkunoglu-Suer et al. [20], previously examined the eight SNPs identified by the GIANT consortium for their association with obesity-related phenotypes at baseline and in response to resistance training in the FAMuSS cohort. They found sex-specific associations with *MC4R* (rs17782313) T>C and BMI; *TMEM18* (rs6548238) C>T and baseline subcutaneous fat volume; and *FTO* (rs9939609) T>A and *SH2B1* (rs7498665) A>G and the subcutaneous fat volume response to resistance training [20].

**Fig. 1:**
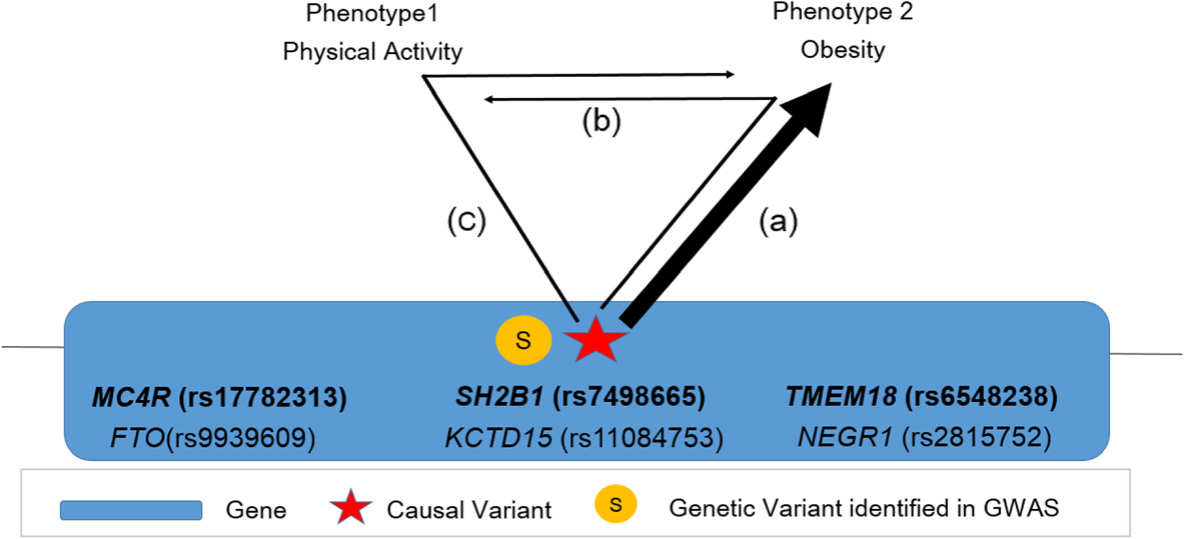
A hypothetical model of the pleiotropy between obesity-related gene loci and physical activity phenotypes. **a**  GWAS-identified gene loci associated with BMI [16]. Two possible explanations supporting the concept of pleiotropy or the shared genetic influence of related phenotypes: **b** An individual with genetic predispositions to obesity is less physically active due to their obesity as discussed by Richmond et al. [33] and others [34, 35]; or (**c**) an individual with genetic predispositions to be less physically active becomes overweight to obese [32, 37]

In our previous work [20] and that of others [15, 17–19], the effect-medication of physical activity on genetic predispositions for overweight and obesity was examined. What was not investigated using this approach is the possibility of obesity-related SNPs influence physical activity behavior (Fig. 1). Pleiotropy refers to the shared genetic influence of related phenotypes [21, 22]. Based upon the concept of pleiotropy, we speculate that the GWAS-identified obesity-related SNPs by the GIANT consortium may associate with physical activity phenotypes. Indeed, there is biological plausibility for doing so as the GWAS SNPs found by the GIANT consortium are expressed in hypothalamus where energy homeostasis is regulated. In addition, recent work in animals has shown that the control of voluntary movement resides in similar central neural pathways as energy intake [23–25]. Therefore, it is plausible that the central nervous system would be an upstream region where these GWAS SNPs share a common biological influence on both obesity and physical activity phenotypes.

Therefore, the purpose of the present study was to investigate the six obesity-related SNPs identified in the GWAS by the GIANT consortium for their association with habitual physical activity that were in Hardy Weinberg Equilibrium in the FAMuSS cohort. We hypothesized that these GWAS obesity-related genetic variants would associate with habitual physical activity.

## Methods

### Overview

This substudy is part of the larger FAMuSS (NIH R01 NS40606-02). FAMuSS was the first systematic study to examine how physiological responses to resistance training are modified by genes and the environment [26]. The institutional review boards from the ten institutions involved with FAMuSS approved the study protocol. All procedures followed were in accordance with the ethical standards of the responsible committee on human experimentation (institutional and national) and with the Helsinki Declaration of 1975, as revised in 2013. Informed consent was obtained from all patients for being included in the study. The experimental design of FAMuSS has been described elsewhere so that only the methods pertaining directly to this substudy will be described in detail [26].

### Subjects

Potential FAMuSS participants were recruited from the eight resistance training sites via strategic flyer placement and in-house listserv and radio announcements. Subjects were excluded if they were <18 or >40 years and if they were taking any medications known to affect skeletal muscle function such as corticosteroids, antihypertensive or anti-lipidemic medications, anabolic steroids, diuretics, arthritis medications (Vioxx, Celebrex), Depo-Provera contraceptive injection, nasal inhalers (Clenbuterol and Rhinocort), lithium, or chronic use of non-steroidal anti-inflammatory drugs. In addition, because the primary aim of FAMuSS was to examine the influence of genetic variation on the muscle size and strength response to resistance training, individuals who reported regular participation in resistance training within the past 12 months prior to enrollment or performed occupational or recreational physical activity that involved heavy use of the upper body were also excluded.

### Body Composition

Anthropometric measures were collected using standardized protocols among the testing sites. Height and weight were measured using a calibrated wall-mounted stadiometer and scale, respectively, from which BMI was calculated (kg/m^2^).

### Physical Activity

Subjects completed the Paffenbarger physical activity questionnaire during their initial visit to assess weekly physical activity over the last year. The Paffenbarger physical activity questionnaire is an eight-item instrument validated among populations similar to the FAMuSS cohort that is used to measure self-reported weekly duration and intensity of physical activity [27, 28]. Physical activity volume in metabolic energy equivalents (MET)-hour/week was derived from the summed time spent (hour/week) times the given MET value for vigorous, moderate, and light intensity physical activity, and sitting and sleeping, respectively [29].

### Genotyping

A sample of whole blood was obtained from each subject, refrigerated, and sent to the Children’s National Medical Center Research (Washington, DC). DNA was isolated from peripheral blood lymphocytes with the Gentra Puregene DNA extraction kit (Qiagen, Valencia, CA) and genotyping was completed using Taqman assays from ABI (Foster City, CA). Allele-specific PCR reactions for each polymorphism included 20 ng DNA, 900 nM forward and reverse PCR primers, 200 nM fluorescent allele discrimination probes (common allele FAM labeled; rare allele VIC labeled), and TaqMan® Universal PCR Master Mix, No AmpErase® UNG (Applied Biosystems, Foster City, CA, USA) in a final volume of 25 μl. The PCR and fluorescent ratio profile was generated after 10 min at 95 °C (denaturation), 44 cycles of 15 s at 92 °C, and 1 min at an annealing temperature of 60 °C. The end point fluorescent readings were analyzed using an ABI 7900HT and the two alleles were called using Sequence Detection System (SDS V 2.3 software; Applied Biosystems, Foster City, CA) and checked manually [30].

In this substudy, 492 subjects were genotyped for the following SNPs identified in the GWAS by the GIANT consortium to associate with BMI that were also in Hardy Weinberg Equilibrium in the FAMuSS cohort (Table 1) (13): *FTO* (rs9939609; *n* = 483) T>A, *KCTD15* (rs11084753; *n* = 490) G>A, *MC4R* (rs17782313; *n* = 480) T>C, *NEGR1* (rs2815752; *n* = 491) A>G, *SH2B1* (rs7498665; *n* = 489) A>G, and *TMEM18* (rs6548238; *n* = 490) C>T.

**Table 1:** Chi-square (*χ* ^2^) and allelic frequencies of single nucleotide polymorphisms examined in the current study

Nearest gene	RefSeq#	Alleles (+/−)	*χ* ^2^	*p*	*q*	*p* value
*FTO*	rs9939609	T/A	0.74	0.40	0.60	0.39
*MC4R*	rs17782313	T/C	0.04	0.21	0.79	0.83
*NEGR1*	rs2815752	A/G	0.71	0.65	0.35	0.39
*SH2B1*	rs7498665	A/G	0.04	0.61	0.39	0.84
*KCTD15*	rs11084753	G/A	0.02	0.66	0.34	0.88
*TMEM18*	rs6548238	C/T	0.08	0.82	0.18	0.78

*FTO* fat mass and obesity-associated gene, *MC4R* Melanocortin 4 receptor, *NEGR1* neuronal growth regulator 1, *KCTD15* potassium channel tetramerization domain containing 15, *SH2B1* Src homology 2 B adapter protein 1, *TMEM18* transmembrane protein 18. df = 1 for all analyses

### Statistical Analysis

Descriptive analyses were performed for all study variables. No SNP was in linkage disequilibrium with the other (*r*^2^ < 0.001). To reveal associations among genotype (dominant/recessive model) and MET-hour/week, multivariable adjusted (age, sex, and BMI) linear regression was used. Then the partial (Type-3) R-square values for the independent variables were computed. In the regression model, log-transformation was considered for the physical activity volume outcome variable to satisfy the underlying assumption (=normality). To estimate the actual MET-hour/week difference among genotypes, the coefficient obtained from the regression model was back transformed (e.g., e^-0.036^ = 0.964), and multiplied by the mean MET-h∙wk^−1^ (i.e., 287.4 MET-hour/week) among the FAMuSS cohort (Table 2). Linear regression analyses were performed using the R Core Team (2015) for Windows (Vienna, Austria); and descriptive statistics were performed using the Statistical Package for the Social Sciences (SPSS) 14.0 for Windows (Chicago, IL, USA). We ran a power calculation based on the R-square difference between the full model vs. the three genetic variants (*MC4R*, *TMEM18, SH2B1*) using Statistical Analysis System (SAS) 9.1.3 for Windows (Cary, NC, USA). Accordingly, we had 92.5 % power with an alpha <0.05 to detect phenotype–genotype differences with three genetic variants and physical activity volume.

**Table 2:** Subject characteristics and self-reported physical activity levels

Characteristics	Total sample	Women	Men
	(*n* = 492)	(*n* = 263)	(*n* = 229)
Age (year)	23.5 ± 0.3	23.2 ± 0.4	23.9 ± 0.4
Body mass index (kg/m)*	24.6 ± 0.2	23.9 ± 0.3	25.4 ± 0.3
Physical activity volume (MET-hour/week)	287.4 ± 2.2	288.5 ± 2.7	286.1 ± 3.7

Values are presented as mean ± standard error

*p = 0.001 (women vs men)

## Results

### Subject Characteristics

The sample consisted of healthy, young European-American men and women (Table 2) with a BMI level that reflected the larger FAMuSS cohort [20, 26], and the general age-matched population from which they were recruited [1]. While age did not differ by sex (*p* > 0.05), men had a higher BMI than women (*p* < 0.001). The self-reported sitting time (6.4 h/day or 40 % of waking time) among this FAMuSS subsample was slightly lower than the average American adults’ sitting time (60 % of waking time) [31].

### Genetic Predictors of Physical Activity Volume (MET-hour/week)

Multivariable linear regression model revealed that *MC4R* (rs17782313) T>C (*p* = 0.02) and *TMEM18* (rs6548238) C>T (*p* = 0.01) were significant predictors of physical activity volume, while *SH2B1* (rs7498665) A>G trended towards significance (*p* = 0.08). *MC4R* (rs17782313) T>C explained 1.1 % (*p* = 0.02), *TMEM18* (rs6548238) C>T explained 1.2 % (*p* = 0.01), and *SH2B1* (rs7498665) A>G explained 0.6 % (*p* = 0.08) of the variability in physical activity volume.

### Genotype and Physical Activity Phenotype Associations

#### *MC4R* (rs17782313) T>C

Subjects with the *MC4R* CC genotype spent 3.5 % less MET-hour/week than T allele carriers (*p* = 0.02). Given the mean value of physical activity volume for the sample (Table 2), a 3.5 % reduction in MET-hour/week among subjects with the *MC4R* CC genotype equates to a 10.1 MET-hour/week decrease in physical activity volume for those with the CC genotype compared to T allele carriers.

#### *TMEM18* (rs6548238) C>T

Subjects with the *TMEM18* T allele spent 4.1 % less MET-hour/week than those with the CC genotype (*p* = 0.01). Given the mean value of physical activity volume for the sample, (Table 2), a 4.1 % reduction in MET-hour/week among *TMEM18* T allele carriers equates to a 11.8 MET-hour/week decrease in physical activity volume for the T allele carriers compared to those with the CC genotype.

#### *SH2B1* (rs7498665) A>G

Finally, subjects with the *SH2B1* GG genotype spent 3.6 % less MET-hour/week than A allele carriers (*p* = 0.08). Given the mean value of physical activity volume for the sample (Table 2), a 3.6 % reduction among subjects with the *SH2B1* GG genotype equates to 10.3 MET-hour/week decrease in physical activity volume for those with the GG genotype compared to A allele carriers.

## Discussion

We tested six SNPs associated with obesity from GWAS by the GIANT consortium for their association with physical activity volume in the FAMuSS cohort. *MC4R* (rs17782313) T>C (*p* = 0.02) and *TMEM18* (rs6548238) C>T (*p* = 0.01) were significant predictors of physical activity volume, while *SH2B1* (rs7498665) A>G trended towards significance (*p* = 0.08). These GWAS obesity-related genotype and physical activity volume associations accounted for ~1 % of the variance in the physical activity volume each. The genotype differences in physical activity volume that we found ranged from 10.1 to 11.8 MET-hour/week (i.e., 730 to 850 kcal/week assuming a mean sample body weight of 72.3 kg) and could theoretically amount to a weight differential of 10.8–12.7 lb per year (i.e., assuming 3500 kcal = 1 lb fat mass). Our results support the notion of genetic pleiotropy or the shared genetic influence among obesity and physical activity phenotypes that should be explored further [22]. Furthermore, the genotype differences we found in weekly physical activity volume have important public health implications for maintaining a healthy weight.

Our finding with *MC4R* (rs17782313) T>C and its association with physical activity is consistent with a prior report that this is an important obesity susceptibility genetic locus that also associates with physical activity. Loos et al. [32] examined whether *MC4R* (rs17782313) T>C influenced self-reported physical activity among parents (*n* = 326, 52 ± 3.4 years) and their offspring (*n* = 343, 28 ± 8.7 years) who were overweight in the Quebec Family Study. The parents with the *MC4R* (rs17782313) CC genotype reported engaging in 986.1 kcal/week less in moderate-to-vigorous sports and recreation than subjects with the CT genotype and 1500.6 kcal/week less than the T allele carriers. Similar to Loos et al. [32], FAMuSS subjects with the *MC4R* (rs17782313) CC genotype spent less weekly physical activity volume than T allele carriers. *TMEM18* (rs6548238) C>T and *SH2B1* (rs7498665) A>G have not yet been examined for their associations with physical activity other than in the FAMuSS cohort, so comparisons to the published literature are not possible.

The biological mechanisms by which SNPs identified in GWAS to associate with obesity-related phenotypes may modulate physical activity are largely unknown. In addition, our findings are based on association and not causation. Nonetheless, as illustrated in Fig. 1, we propose two plausible explanations for the associations we observed: 1) an individual with genetic predispositions to obesity [16] is less physically active due to their obesity as discussed by Richmond et al [33] and others [34, 35] (Fig. 1b); or 2) an individual with genetic predispositions to be less physically active becomes overweight to obese (Fig. 1c). Although we acknowledge both explanations are possible, the biological features of the SNPs we examined appear to support the second explanation. In addition, Orkunoglu-Suer et al. [20] found that the same three SNPs that associated with physical activity volume in our study [i.e., *MC4R* (rs17782313) T>C, *TMEM18* (rs6548238) C>T, and *SH2B1* (rs7498665) A>G] also associated with body composition phenotypes including BMI among the FAMuSS cohort further substantiating the concept of pleiotropy and our hypothesis of the essential role that physical activity has in mediating overweight and obesity.

It is interesting to note that the three SNPs that we and Okkunoglu-Suer et al. [20] examined are expressed in brain and/or hypothalamus where energy homeostasis is regulated [16]. The hypothalamus is the primary output node for the limbic system, which is responsible for endocrine function and behavior reinforcement. The limbic system is implicated in the control of food procurement as an evolutionarily conserved survival mechanism to defend against famine [36]. In this regard, these three GIANT consortium identified GWAS obesity SNPs that we found to be associated with physical activity and Orkunoglu-Suer et al. found to be associated with body composition phenotypes [20] have been classified as hyperphagic genes related to appetite suppression and satiety whose regulation resides in the dopaminergic projection from the limbic system [37–39]. Recent research in animals suggests that the ‘pleasure-reward’ system residing in the dopaminergic pathway that regulates appetite and satiety has a key role in voluntary movement [25, 40] and heightened reward sensitivity in animals with obesity that binge eat [41, 42]. The apparent shared genetic influence of energy intake and expenditure whose regulation resides in similar central nervous system pathways, particularly in the hypothalamus and dopaminergic pathway, is noteworthy, supports our hypothesis, and merits further investigation.

This study has several limitations as this was a substudy of FAMuSS whose primary purpose was to examine the influence of genetic variation on the muscle size and strength response to resistance training. The FAMuSS cohort consisted primarily of healthy, European-American young adults with the characteristics of the subjects in this substudy mirroring those of the larger cohort (Table 3). The physical activity data we collected with the Paffenbarger physical activity questionnaire were susceptible to subject recall and social desirability bias [43], and there were no measures of reproducibility and validity about the Paffenbarger’s questionnaire in our sample. However, the Paffenbarger physical activity questionnaire is a well validated and reliable method for assessing leisure time physical activity in similar populations to the present study [44]. Our only measure of body composition was BMI which does not discriminate among body fat, muscle mass, or bone [45]. Last, we did not measure energy intake nor did we obtain physiological data that would provide insight into mechanisms for the genotype–physical activity phenotype associations we found.

**Table 3:** Genotype frequencies of single nucleotide polymorphisms for FAMuSS participants

Gene	RefSeq#	Alleles (+/−)	Obesity risk allele^a^	Obesity risk allele frequency in FAMuSS (+)	Published obesity risk allele frequency for CEU (+)^b^
*MC4R*	rs17782313	T/C	C	0.790	0.735
*TMEM18*	rs6548238	C/T	C	0.824	0.850
*FTO*	rs9939609	T/A	A	0.395	0.460
*NEGR1*	rs2815752	A/G	A	0.650	0.637
*SH2B1*	rs7498665	A/G	G	0.392	0.381
*KCTD15*	rs11084753	G/A	G	0.664	0.690

*CEU* European Caucasian, *SNPs* single nucleotide polymorphisms, *FAMuSS* Functional SNPs Associated with Muscle Size and Strength, *MC4R* melanocortin 4 receptor, *TMEM18* transmembrane protein 18, *FTO* fat mass and obesity-associated gene, *NEGR1* neuronal growth regulator 1, *SH2B1* Src homology 2B adapter protein 1, *KCTD15* potassium channel tetramerization domain containing 15

^a^According to Willer et al*.* [16]

^b^Utah residents with northern western European ancestry from the Center for the Study of Human Polymorphisms (CEPH) collection used in haplotype map (HapMap)

Yet, this study has several important strengths. FAMuSS is recognized as one of the largest exercise genomics studies ever conducted [46]. In addition, physical activity is more heritable among young than older adults [47]. Therefore, as the average age of the FAMuSS subjects was 24 years, the heritability of their habitual physical activity levels was not confounded with the influence of age that may have contributed to us finding the genotype–physical activity phenotype associations we observed.

## Conclusion

In summary, we have shown that three genetic variants associated with obesity in GWAS by the GIANT consortium also associated with habitual physical activity in the FAMuSS cohort. These SNPs accounted for ~1 % of the variance in physical activity levels each. The genotype differences in physical activity volume we found ranged from 10.1 to 11.8 MET-hour/week equating to a potential weight differential of 10.8–12.7 lb annually. Our findings suggest obesity and physical activity have a shared genetic influence that is regulated by common central neural pathways that merit further investigation.
